# Supplementary figures and images for: Overall Observed Survival of Female Breast, Cervical, Colorectal, and Prostate Cancers in Antigua and Barbuda, 2017–2021: Retrospective Data from Four Study Sites
Source: Int J Environ Res Public Health. 2025 Feb 7;22(2):235. doi: 10.3390/ijerph22020235 (PMC11855306; doi:10.3390/ijerph22020235)

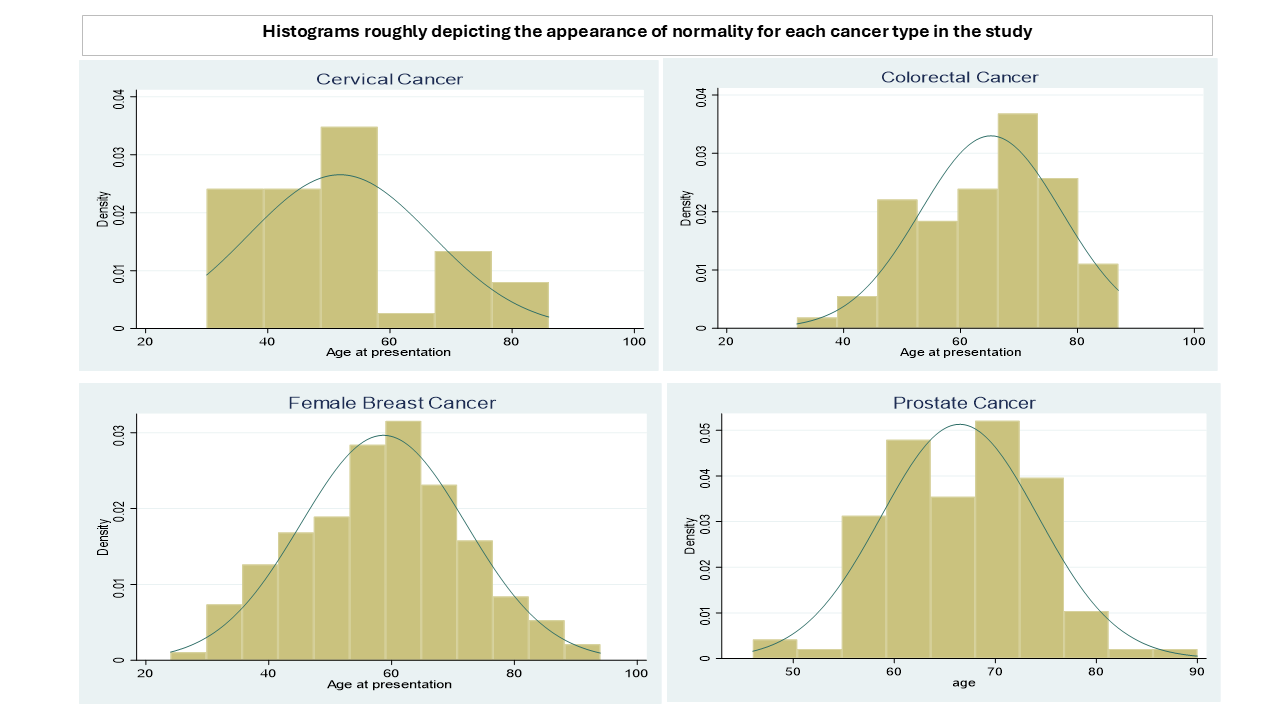

Supplement: Supplementary file 1 [file ijerph-22-00235-s001.zip › Supplementary file 1.tif]

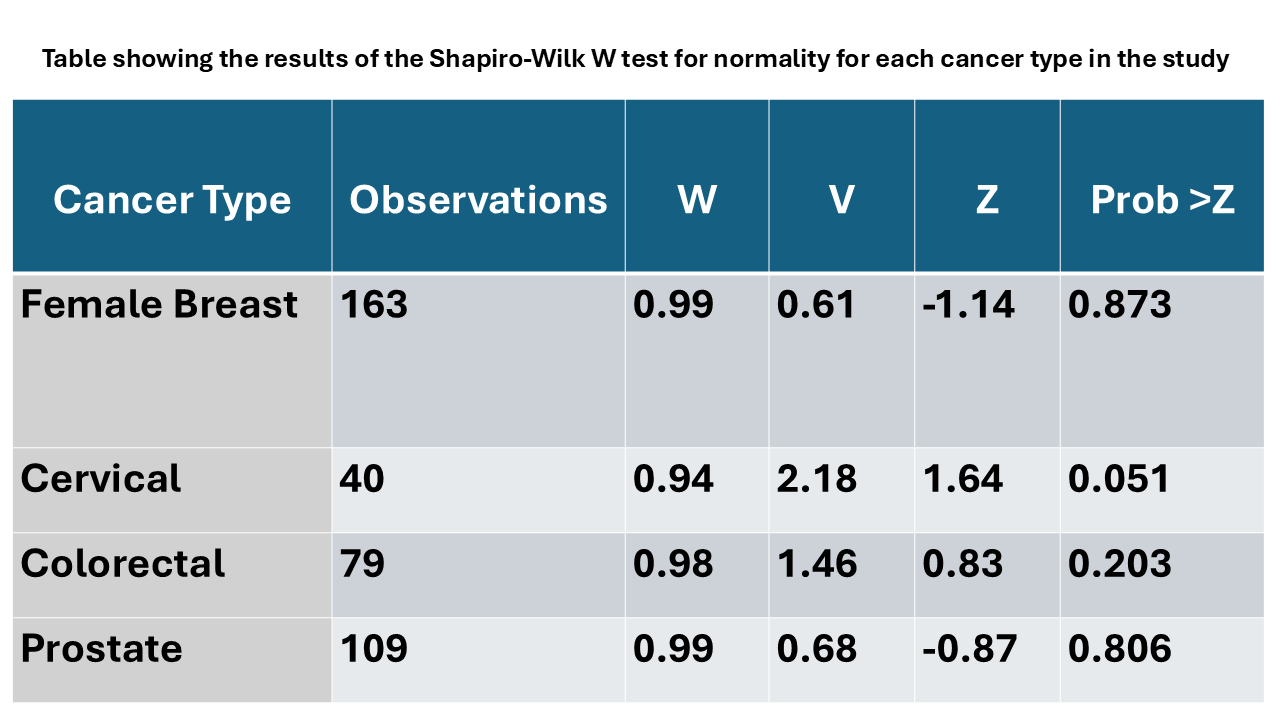

Supplement: Supplementary file 1 [file ijerph-22-00235-s001.zip › Supplementary file 2.tif]
